# Anti-apoptotic and Immunomodulatory Effect of CB2 Agonist, JWH133, in a Neonatal Rat Model of Hypoxic-Ischemic Encephalopathy

**DOI:** 10.3389/fped.2020.00065

**Published:** 2020-02-27

**Authors:** Bhavna Gupta, Mary G. Hornick, Seema Briyal, Ramona Donovan, Preetha Prazad, Anil Gulati

**Affiliations:** ^1^Division of Neonatology, Department of Pediatrics, Advocate Children's Hospital, Park Ridge, IL, United States; ^2^Chicago College of Pharmacy, Midwestern University, Downers Grove, IL, United States; ^3^Advocate Center for Pediatric Research, Advocate Children's Hospital, Park Ridge, IL, United States

**Keywords:** neonatal, encephalopathy, hypoxic ischemia, neuroprotection, CB2 agonist

## Abstract

**Introduction:** Neonatal HIE is associated with high morbidity and mortality. Current research, is focused on developing alternative treatments to therapeutic hypothermia for treatment of HIE. The endocannabinoid system is known to be influential in neuronal protection. Activation of brain CB2 receptors, has been shown to reduce inflammatory markers and decrease infarct volume in adult cerebral ischemic models.

**Methods:** Rat pups were divided into six groups: 1—Placebo; 2—JWH133; 3—HIE + Placebo; 4—HIE + JWH133; 5—HIE + Hypothermia + Placebo; and 6—HIE + Hypothermia + JWH133. HIE was induced in in groups 3–6 by right carotid ligation on postnatal day 7 followed by placement in a hypoxic chamber. Pups in groups 5 and 6 were treated with hypothermia. Western blot analysis was used to analyze brain tissue for acute inflammatory markers (IL-6, TNFα, MIP1α, and RANTES), immunoregulatory cytokines (TGFβ and IL-10), and CB2 receptor expression. DNA fragmentation in the brains of pups was determined via TUNEL staining post HIE.

**Results:** The combination of JWH133 and hypothermia significantly reduced tumor necrosis factor α (TNFα) (−57.7%, *P* = 0.0072) and macrophage inflammatory protein 1α (MIP1α) (−50.0%, *P* = 0.0211) as compared to placebo. DNA fragmentation was also significantly reduced, with 6.9 ± 1.4% TUNEL+ cells in HIE+JWH133 and 12.9 ± 2.2% in HIE+Hypothermia + JWH133 vs. 16.6 ± 1.9% in HIE alone. No significant difference was noted between groups for the expression of interleukins 6 and 10, RANTES, or TGFβ. After 8 h, CB2 receptor expression increased nearly 2-fold in the HIE and HIE + JWH133 groups (+214%, *P* = 0.0102 and +198%, *P* = 0.0209, respectively) over placebo with no significant change in the hypothermia groups. By 24 h post HIE, CB2 receptor expression was elevated over five times that of placebo in the HIE (*P* < 0.0001) and HIE + JWH133 (*P* = 0.0002) groups, whereas hypothermia treatment maintained expression similar to that of placebo animals.

**Conclusion:** These results indicate that the combination of CB2 agonist and hypothermia may be neuroprotective in treating HIE, opening the door for further studies to examine alternative or adjuvant therapies to hypothermia.

## Introduction

Neonatal hypoxic ischemic encephalopathy (HIE) is the second most preventable cause of neuro disability worldwide (1). In 2010, 8.5 cases per 1,000 live births were estimated to have developed neonatal encephalopathy associated with intrapartum events (2). Due to the high morbidity and mortality associated with HIE, it has been an area of global health concern. In 2010, the Eunice Kennedy Shriver National Institute of Child Health and Human Development (NICHD) invited a panel of experts to review the available evidence, identify knowledge gaps, and suggest research priorities. The panel suggested the need for the development of adjuvant therapies to hypothermia, the development of biomarkers, as well as further refinements of hypothermia therapy to further protect the brain and improve the outcomes of these children (3).

Therapeutic hypothermia is currently the standard of care for infants who have experienced perinatal asphyxia (PA) leading to HIE. Several trials such as the ICE study (4), Cool Cap study (5), NICHD study (6), and TOBY study (7) have shown overall benefit of hypothermia in the reduction of neurological injury caused by PA. Unfortunately, not all NICUs are equipped to provide therapeutic hypothermia. Unavailability of trained personnel, equipment, and pediatric neurology support prohibit hypothermia treatment in many NICUs. Moreover, >40% of neonates undergoing hypothermia still develop adverse neurological outcomes (8). While clinical trials report follow-up data on children treated with hypothermia for only up to 24 months (4, 6, 8), the long term neurodevelopmental impact of this therapy remains unclear (9).

One of the key cellular pathways leading to neuronal injury in HIE is inflammation. Hypoxic insult leads to activation of the inflammatory cascade involving cytokines (10, 11), accumulation of inflammatory cells (12), and chemokines contributing to neuronal injury (11, 13, 14). Microglial cells are resident macrophage cells in the brain and constitute the first line of defense by releasing cytokines and recruiting leucocytes. Studies in rat models have reported a peak in inflammatory markers, tumor necrosis factor alpha (TNF α) (15) and interleukin 6 [IL6] as early as 6- 12 h following insult which corresponds to the peak of neuronal apoptosis (10). This is followed by peak in beta chemokine, macrophage inflammatory protein 1 alpha (MIP1 α) at 6–72 h. Induction of markers like Regulated on activation, normal T cell expressed and secreted (RANTES) preceded influx of macrophages and lymphocytes to the infarct area (15). Transforming growth factor beta (TGF β) and Interleukin 10 (IL 10) are the pro inflammatory cytokines for microglial cells and macrophages.

The endocannabinoid system is an intricate neuro-modulatory system in human body consisting of two cannabinoid (CB) receptors (16); cannabinoid receptor type 1 and cannabinoid receptor type 2 (CB1 and CB2), as well as their endogenous ligands named endocannabinoids and enzymes responsible for their synthesis and degradation (17). Recent evidence suggests that one of the main physiological functions of this system is to modulate neuroinflammation. Cannabinoid receptors can be found in neural and non-neural tissues throughout the body. Compared to CB1, CB2 is highly expressed in immune cells in peripheral tissues and the central nervous system (CNS) (16). Use of a CB2 agonist has been shown to effectively reduce infarct volume and inflammatory markers in the brain (12).

Cannabidiol (CBD), the main non-psychoactive component of Cannabis sativa, alone, or as an adjunct to hypothermia has demonstrated reduction in excitatory, inflammatory, and oxidative stress markers in animal models of HIE (11). Anti-inflammatory/anti-excitatory effects of CBD are mediated mainly by CB2 (13). Given that the anti-excitatory and anti-inflammatory effects of CBD, a non-selective ligand for the CB receptors, were mediated by activation of the CB2 receptor, we hypothesized that selective CB2 activation may have similar or more potent effects. JWH 133 is a potent selective CB2 agonist that has 200-fold selectivity over CB1 receptors (Ki or inhibitor constant values are 3.4 and 677 nM, respectively).

The goal of the present study was to determine whether a selective CB2 agonist would have a neuro protective effect with or without hypothermia in a neonatal HIE rat model by reducing the inflammatory reaction. Acute inflammatory markers (IL6, TNF α, MIP1 α, RANTES) were measured at 8 and 24 h following injury to capture the various post-injury peaks previously reported (15). The effect of the CB2 agonist on the response of immunomodulatory microglial cells was determined by measuring the expression of macrophage phenotype markers, TGF β, and IL 10.

## Materials and Methods

### Animals

**Figure 1:**
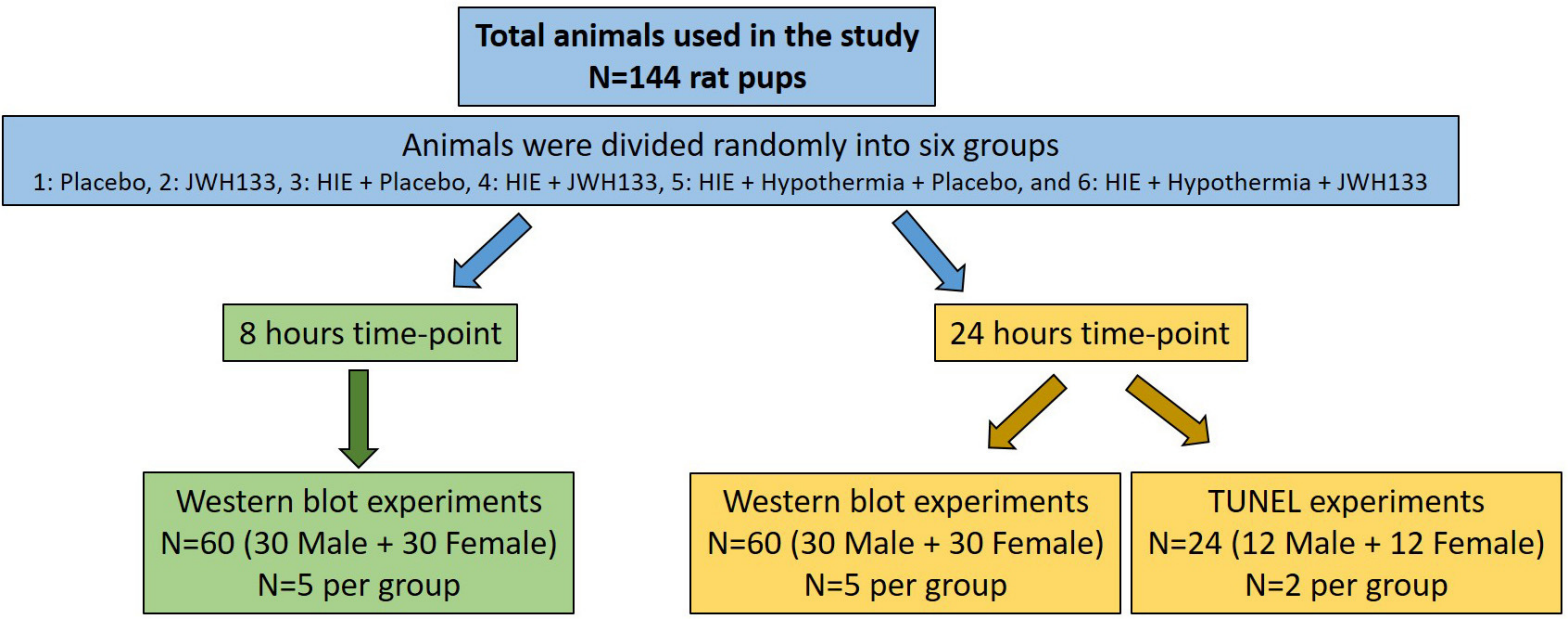
Overall experimental design of the study.

Timed pregnant Sprague-Dawley dams (Envigo, Indianapolis, IN) were housed singly in a room with controlled light (6:00 a.m.−6:00 p.m.), humidity (50 ± 10%), and temperature (23 ± 1°C), with food and water available *ad libitum*. Dams were allowed to give birth naturally. A total of 144 pups were used in the study. For western blot experiments: 5 animals per group (6 groups × 2 genders × 2 time-points = 120 animals (60 Male and 60 Female) and for TUNEL experiments: 2 animals per group (6 groups × 2 genders × 1 time-point = 24 animals (12 Male and 12 Female) were used. Pups were divided randomly on postnatal day (PND) 7 into six groups: 1–Placebo, 2–JWH133, 3–HIE + Placebo, 4–HIE + JWH133, 5–HIE + Hypothermia + Placebo, and 6–HIE + Hypothermia + JWH133 (Figure 1). Animal care, anesthesia, and surgical procedures were performed in accordance with the guidelines for animal care and use following approval by the Institutional Animal Care and Use Committee (IACUC, MWU 2665) of Midwestern University. Dams and pups were monitored for overall appearance, failure to thrive, and any neurological defects from birth until the end of the experiment.

### Induction of Hypoxic-Ischemic Encephalopathy

Pups in groups 3–6 were subjected to hypoxic-ischemic encephalopathy via unilateral carotid ligation followed by a low oxygen environment on PND 7. Briefly, pups were anesthetized with 5% inhaled isoflurane (Henry Schein Animal Health, Dublin, OH) followed by 2% for maintenance. The right common carotid artery was carefully isolated from the vagus nerve and ligated. The wound was closed with surgical glue and the pups allowed to recover on a neoprene pad with controlled temperature of 37°C for 20 min. Pups were then placed in a hypoxic chamber (92% N, 8% O_2_) for 120 min. During the hypoxia procedure, surface body temperature was controlled in all the animals using neoprene pad with controlled temperature of 37°C. Pups were again allowed to recover for 20 min and administered placebo (saline) or JWH133, after which pups in groups 3 and 4 were reintroduced to their dams at room temperature.

### Drugs

Following unilateral carotid ligation and hypoxia, pups in groups 1, 3, and 5 were administered placebo (1:19; EtOH: 0.9% saline) intraperitoneally (IP). The CB2 agonist JWH133 (Tocris Bioscience, Minneapolis, MN) was dissolved in 1:19 EtOH: Saline and administered to pups in groups 2, 4, and 6 at a dose of 1.5 mg/kg, IP, following carotid ligation and hypoxia. The dose of JWH133 was chosen based on a previous study which reported that 1.5 mg/kg JWH133 was found to reduce infarct volume along with inflammatory markers in an adult model of cerebral ischemia.

### Hypothermia Treatment

Pups in groups 5 and 6 were placed on a neoprene pad connected to a ThermaZone® Hot/Cold Therapy pump following the 120 min hypoxia, drug administration and 20 min recovery period. The body temperature of the pups was lowered over a 15 min period and maintained at 33 ± 1°C for 4 h. Pups were then rewarmed to 37 ± 1°C and placed with their respective dams at room temperature.

### DNA Fragmentation

DNA fragmentation, a measure of apoptosis, in brains of rat pups was determined via terminal deoxynucleotidal transferase dUTP nick end labeling (TUNEL) at 24 h post HIE. Brains were fixed with 4% paraformaldehyde and sliced into 10 μm-thick sections using a cryostat (Microtome HM 505E, Walldorf, Germany). Brain tissue sections were processed using the Click-iT™ TUNEL Alexa Fluor™ 488 Imaging Assay according to the manufacturer's instructions (Invitrogen, Carlsbad, CA). Brain cortical cells positively stained for TUNEL DNA fragmentation were quantified after imaging through Nikon TiE inverted fluorescent microscope with 60x objective. Captured microscopic images were processed with NIS-Elements AR 4.13 software (Nikon, Melville, NY) and TUNEL positive nuclei were marked and counted. The data was represented as percent TUNEL positive cells normalized to the total number of nuclei in each field.

### Protein Expression

Five male and five female pups from each group were euthanized via decapitation at 8 or 24 h post HIE. Whole brains were dissected out, washed with saline, and stored at −80°C for Western blot analysis. The brains were homogenized in RIPA buffer and protein concentration of each sample was determined as previously described. Solubilized protein (60 μg) was denatured in Laemmli sample buffer, resolved in 10% SDS-PAGE, and transferred onto a nitrocellulose membrane. Primary antibodies (1:1,000, Abcam, Cambridge, MA) consisted of anti-TGF beta 1 (ab92486), anti-macrophage inflammatory protein 1 alpha/CCL3 (ab25128), anti-TNF alpha (ab6671), anti-RANTES (ab9783), anti-IL10 (ab9969), anti-IL6 (ab9324), and anti-cannabinoid receptor II (ab3561). β-actin (1:10,000; Sigma Aldrich, St. Louis, MO; A1978) was used as a loading control. Proteins labeled with SuperSignal West Pico Chemiluminescent Substrate (Thermo Scientific, Waltham, MA) were imaged with a BioRad Chemidoc Imaging System (BioRad, Hercules, CA). Protein expression was analyzed with ImageJ (NIH) software and expressed as a ratio between the protein of interest and β-actin.

### Data Analysis

A power analysis, conducted with GraphPad Instat-2.00, indicated that a sample size of 8 per group (4 male, 4 female) was sufficient to achieve a power of 80% and a level of significance of 0.05. Ten animals (5 male, 5 female) per group were used in the present study. Based on previous studies, an estimated effect size of 20% change in protein levels was utilized for the power analysis (5). Data was analyzed using GraphPad Prism 8 software (GraphPad, San Diego, CA). Intergroup comparison of DNA fragmentation and protein expression were determined by one-way analysis of variance (ANOVA) followed by Tukey's multiple comparisons test. P values of <0.05 were considered significant.

## Results

### DNA Fragmentation

**Figure 2:**
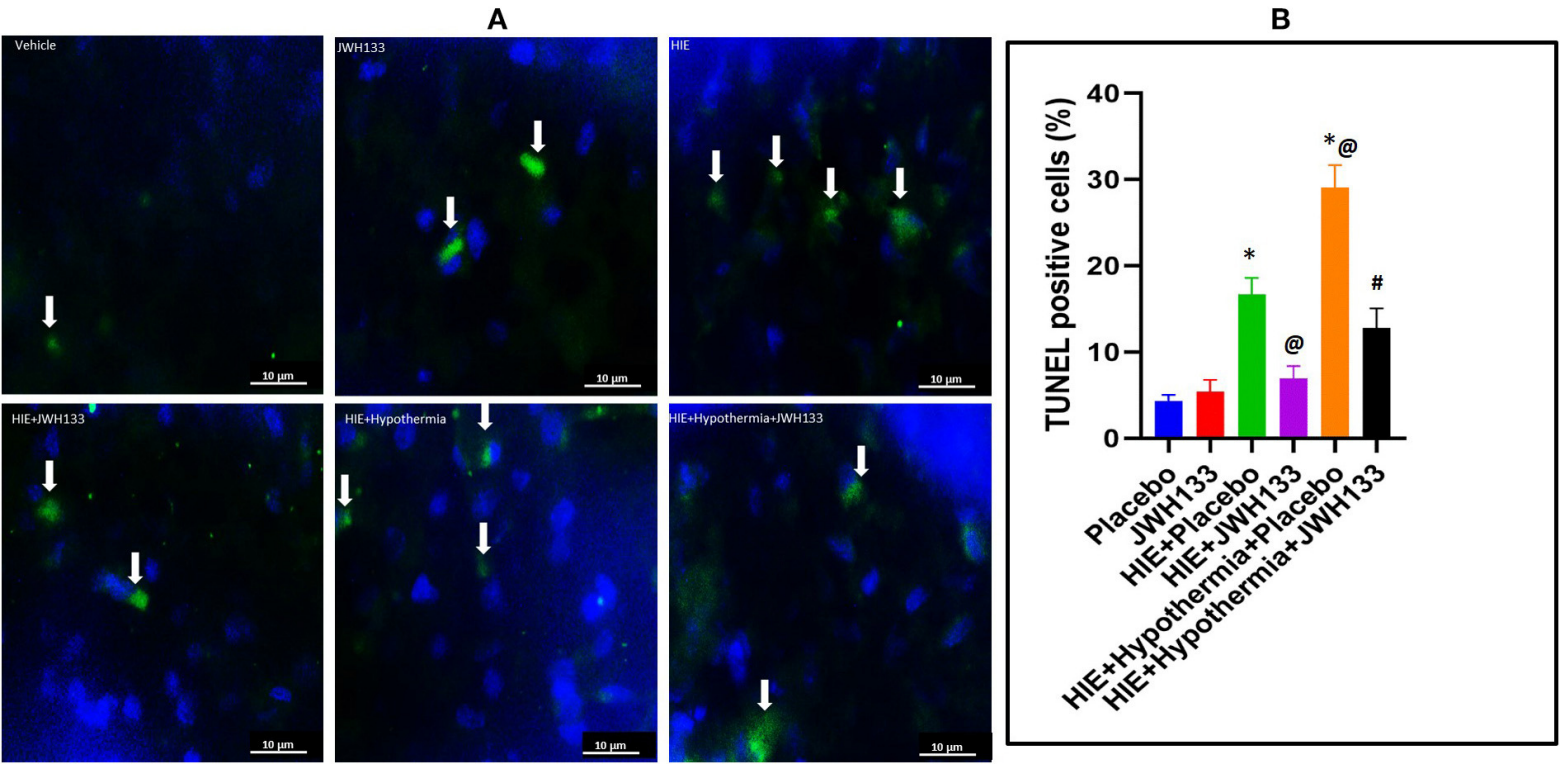
DNA fragmentation detection in the rat brain following HI. **(A)** Representative images of TUNEL staining for DNA fragmentation (apoptosis) in the experimental groups. Arrows indicate TUNEL+ cells. **(B)** Graph represents percent TUNEL positive cells in the rat brain 7 days post HI. ^*^P < 0.001 vs. placebo; ^@^*P* < 0.05 vs. HIE + placebo; ^#^*P* < 0.0001 vs. HIE + Hypothermia + Placebo.

DNA fragmentation, a hallmark of apoptosis, was significantly increased following HIE. The analyzed brain tissues of rat pups not subjected to ischemia/hypoxia was seen with 4.3 ± 0.7% TUNEL+ cells, while brain tissues of pups subjected to HIE + Placebo had 16.6 ± 1.9% TUNEL+ cells (Figure 2; *P* = 0.0008). Treatment of HIE with JWH133 alone and in conjunction with hypothermia, significantly reduced the number of apoptotic cells (6.9 ± 1.4% and 12.9 ± 2.2%, TUNEL+ cells, respectively). We have also observed a significant increase in TUNEL+ cells in HIE with hypothermia compared to HIE alone (29.1 ± 2.6% TUNEL+ cells, *P* = 0.0008). Moreover, there was significant reduction of DNA fragmentation in HIE with JWH133 in conjunction with hypothermia compared to HIE + Hypothermia + Placebo group (*p* < 0.0001). These results indicate that the CB2 receptor agonist, both alone and when combined with hypothermia, is capable of reducing apoptosis significantly, in the cortical region of the neonatal brain following hypoxia.

### Acute Inflammatory Markers

**Figure 3:**
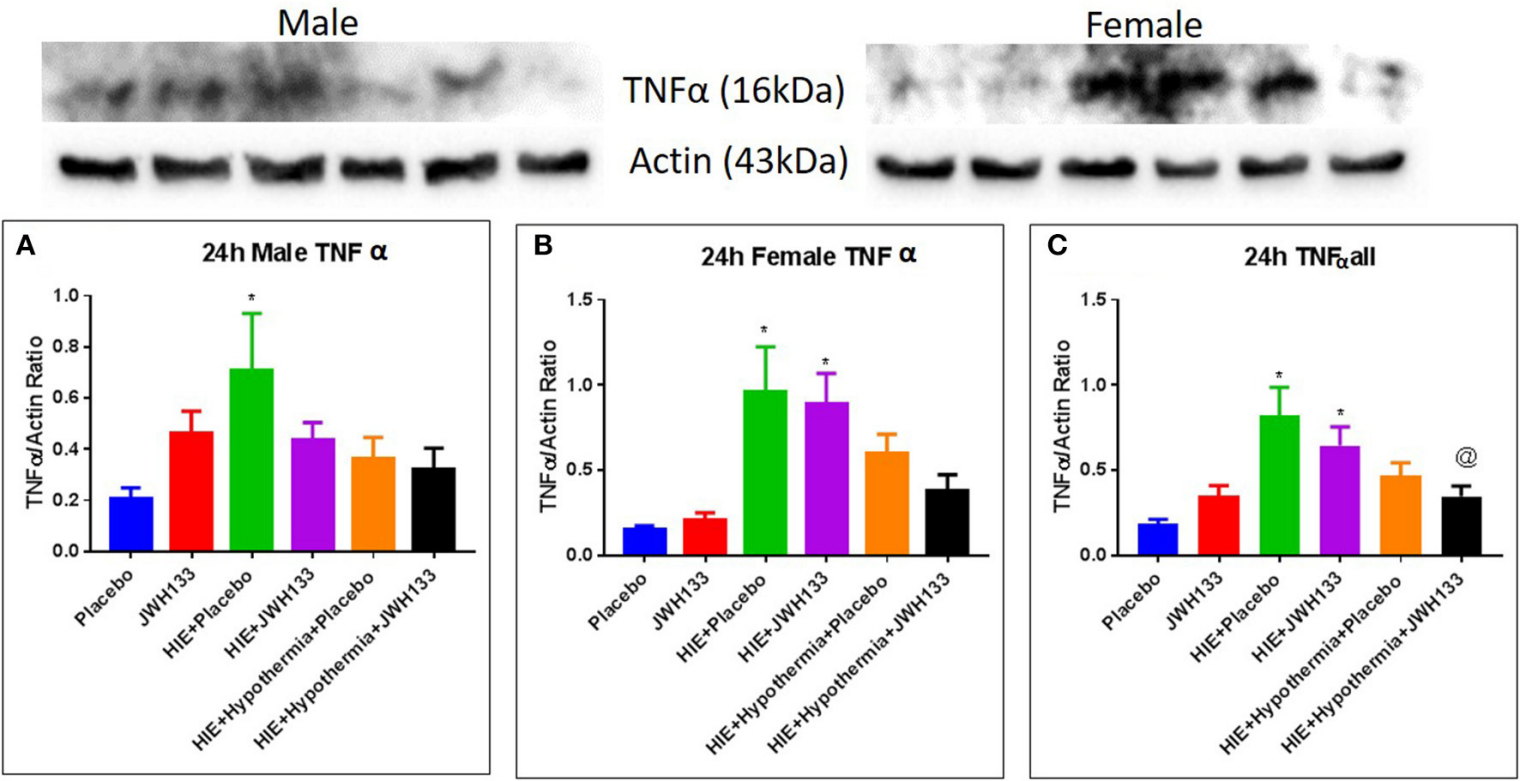
Western blots and densitometry graphs of “Tumor necrosis factor α” (TNF α) in rat brain at 24 h after HI. **(A)** TNF α levels in male pups at 24 h. **(B)** TNF α levels in female pups at 24 h. **(C)** Mean TNF α levels of both male and female pups combined. Actin was used as a loading control. ^*^P < 0.05 vs. placebo; ^@^*P* < 0.05 vs. HIE + placebo.

The expression of acute inflammatory markers—interleukin 6 (IL-6), tumor necrosis factor alpha (TNFα), macrophage inflammatory protein 1 alpha (MIP-1a) and RANTES—was determined in whole brain homogenate at 8 and 24 h post HIE induction. Eight hours after hypoxia, no significant difference was detected between control, untreated-, or treated-HIE groups in any of the acute inflammatory markers. Likewise, expression of IL-6 remained unaltered in any of the groups at 24 h post HIE. Expression of TNFα was increased in both male and female HIE + Placebo groups [Figure 3, *P* = 0.0256, *F*_(5, 29)_ = 2.513 and *P* = 0.0093, *F*_(5, 22)_ = 6.032, respectively], while only the female (*P* = 0.0128) but not the male HIE + JWH133 animals had increased expression of TNFα over that of non-HIE animals at 24 h following hypoxia and ischemia. Combining male and female pups, TNFα expression was elevated in both HIE + Placebo [*P* = 0.0002, *F*_(5, 57)_ = 6.191] and HIE + JWH133 (*P* = 0.0074) compared with non-HIE. Treatment with JWH133 + hypothermia, on the other hand, significantly reduced TNFα expression (−57.7%, *P* = 0.0072) as compared to HIE alone.

**Figure 4:**
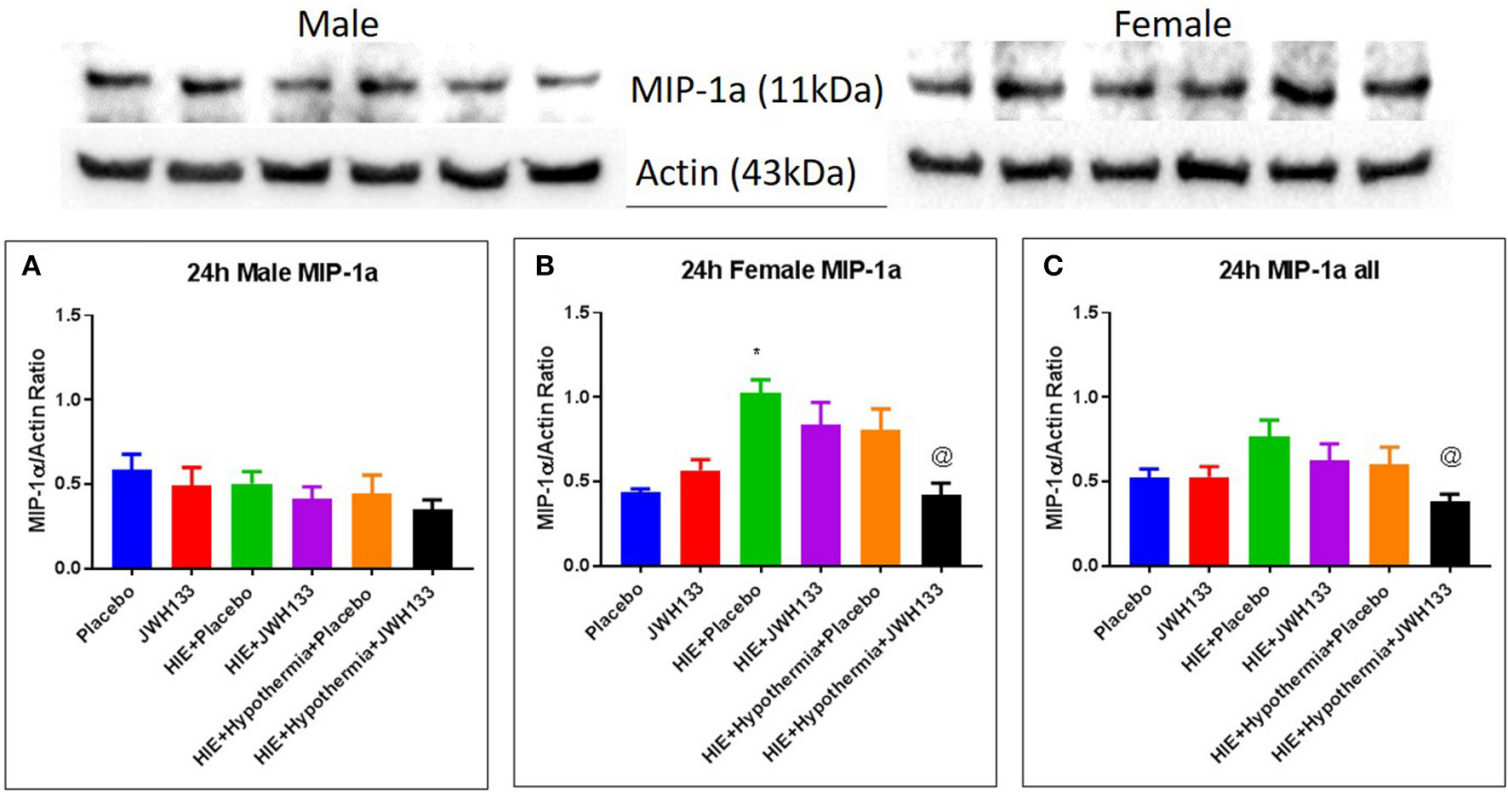
Western blots and densitometry graphs of “macrophage inflammatory peptide 1α” (MIP 1α) in rat brain 24 h after HI. **(A)** MIP 1α levels in male pups at 24 h. **(B)** MIP 1α levels in female pups at 24 h. **(C)** Mean MIP 1α levels in both male and female pups combined. Actin was used as a loading control. ^*^P < 0.05 vs. placebo; ^@^*P* < 0.05 vs. HIE + placebo.

**Figure 5:**
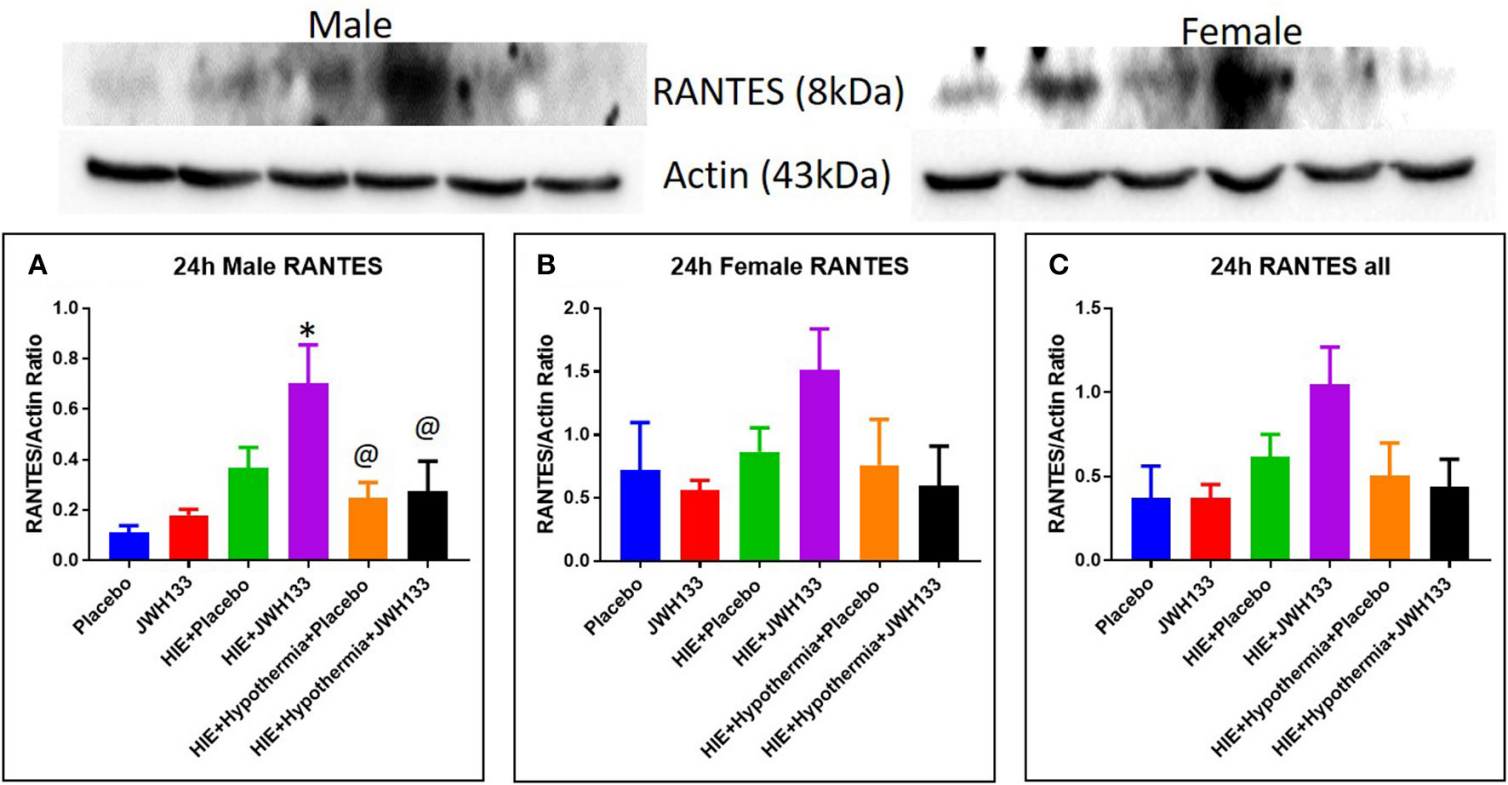
Western blots and densitometry graphs of “regulated on activation normal T cell expressed and secreted” (RANTES) in rat brain 24 h after HI. **(A)** RANTES levels in male pups at 24 h. **(B)** RANTES levels in female pups at 24 h. **(C)** Mean RANTES levels in both male and female pups combined. Actin was used as a loading control. ^*^P < 0.05 vs. placebo; ^@^*P* < 0.05 vs. HIE + JWH 133.

Expression of MIP-1α at 24 h post hypoxia was increased in female [Figure 4, *P* = 0.0030, *F*_(5, 20)_ = 6.746] but not male HIE + Placebo pups as compared to non-HIE. Similarly to TNFα, the combination of JWH133 and hypothermia reduced this increased expression (−58.6%, *P* = 0.0024 for female pups and −50.0%, *P* = 0.0211 for female and male pups combined). Unlike TNFα and MIP-1α, the expression of RANTES was not significantly increased in HIE + Placebo groups at 24 h following HIE. In male pups, however, RANTES expression was increased in the HIE + JWH133 group [Figure 5, *P* = 0.0026, *F*_(5, 18)_ = 5.301] compared to non-HIE pups, while hypothermia alone and in conjunction with JWH133 reversed this elevated expression (*P* = 0.0237 and *P* = 0.0377, respectively). Overall, neither JWH133 nor hypothermia alone significantly reduced the expression of acute inflammatory markers which were elevated as a result of HIE. The combination of JWH133 and hypothermia treatment, however, did significantly reverse HIE-induced inflammation indicating that CB2 receptor agonists may be a novel adjuvant therapy to hypothermia.

### Immunoregulatory Cytokines

The expression of immunoregulatory/immunosuppressive cytokines transforming growth factor beta (TGFβ) and interleukin 10 (IL-10) was determined in whole brain homogenate at 8 and 24 h following the induction of HIE. Neither TGFβ nor IL-10 expression was significantly altered by HIE or treatment with either JWH133 ± hypothermia at 8 or 24 h in male or female pups.

### Cannabinoid Receptor II Expression

**Figure 6:**
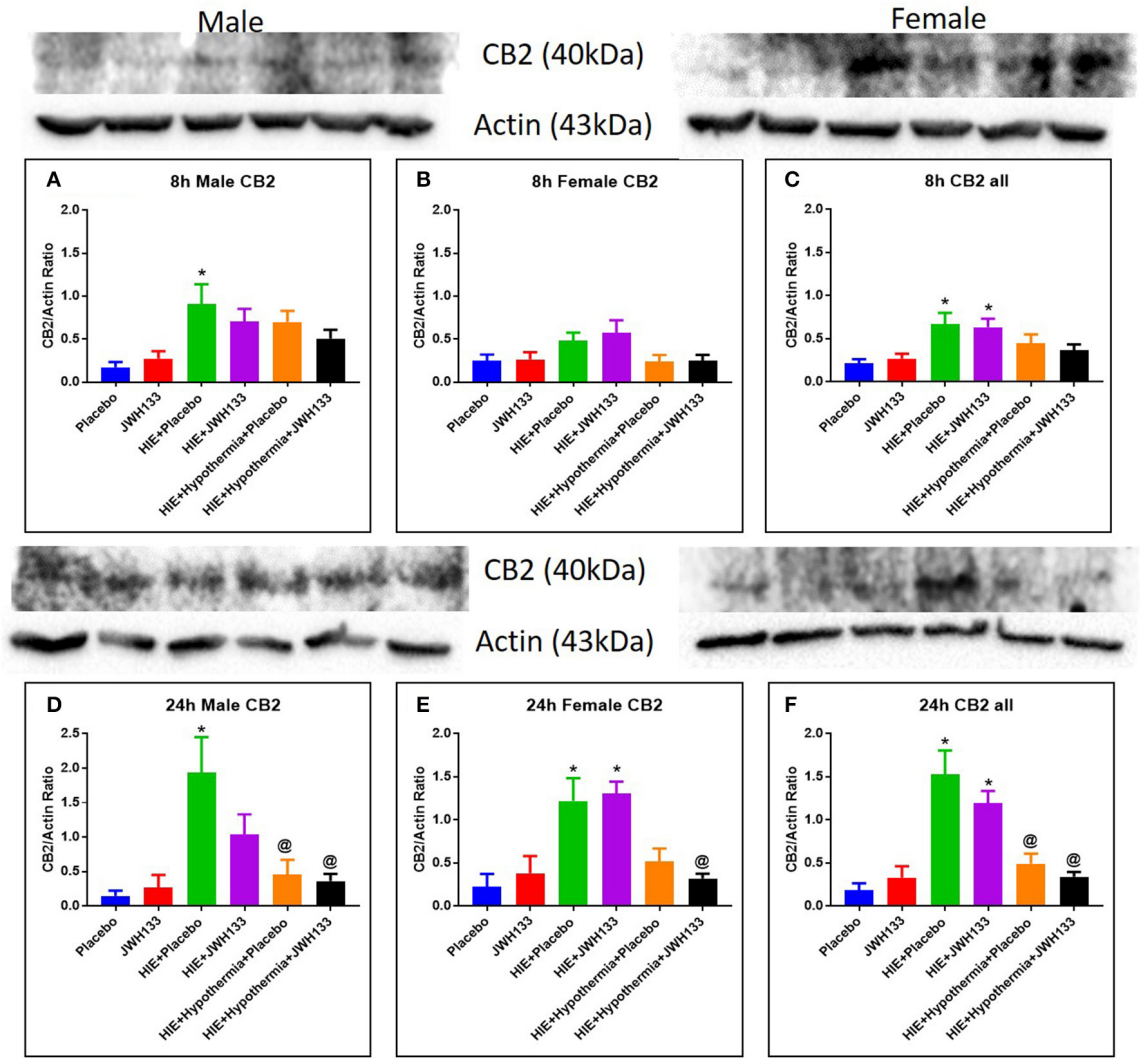
Western blots and densitometry graphs of Cannabinoid receptor 2 (CB2) expression in the rat brain 8 h after HI: **(A)** Males, **(B)** Females. **(C)** Combined. Cannabinoid receptor 2 (CB2) expression in the rat brain 24 h after HI: **(D)** Males. **(E)** Females. **(F)** Combined. Actin was used as a loading control. ^*^P < 0.05 vs. placebo; ^@^*P* < 0.05 vs. HIE + placebo.

The expression of CB2 receptors in whole brain homogenates was determined as described above for the acute inflammatory markers and immunoregulatory cytokines. At 8 h after the induction of HIE, CB2 receptor expression was significantly elevated in both HIE + Placebo and HIE + JWH133 groups [Figure 6, *P* = 0.0102 and *P* = 0.0209, *F*_(5, 48)_ = 4.337] as compared with the non-HIE placebo group. CB2 receptor expression was further elevated in these groups [*P* < 0.0001 and *P* = 0.0002, *F*_(5, 38)_ = 13.75] at 24 h post HIE in both male and female pups. Treatment with hypothermia alone (*P* = 0.0003) and in combination with JWH133 (*P* < 0.0001) significantly reduced this increase in CB2 receptor expression following neonatal HIE. These findings show that HIE does have a significant impact on the expression of the CB2 receptor, which can be negated with the use of hypothermia therapy.

## Discussion

HIE continues to be one of the major preventable causes of neurodisability. Hypothermia, the current gold standard of treatment due to its limited availability and technical complications is not an easily accessible and 100% curative therapy. There is definitely need for adjuvant therapies that are easily accessible and have additive neuroprotective effect.

HIE is known to cause damage by initiating the inflammatory cascade, eventually leading to neuronal death. Cannabidiol (CBD) alone and in conjugation with hypothermia has been shown to decrease the percentage of damaged neurons (11, 14). This effect was attenuated in the presence of a CB2 receptor antagonist (14). *In vitro* studies in newborn mice show an anti-apoptotic effect of CBD, and this effect was abolished in presence of CB2 receptor antagonist (13). In our study JWH 133, a selective CB2 agonist, shows its neuroprotective effect by its anti-apoptotic and anti-inflammatory action in conjugation with hypothermia.

Tumor necrosis factor alpha (TNFα) levels peak at 4–6 h following hypoxic ischemic injury (11, 18) with some elevation at 24 h (18). In our study, TNFα was significantly elevated at 24 h in the HIE group. We did not observe a significant peak at 8 h. Treatment with both hypothermia and JWH 133 was effective in reducing TNFα levels at 24 h. Hypothermia alone has shown to decrease acute inflammatory marker TNFα at 6 h (11) and this is potentiated by use of CBD (11, 13). In our study JWH133 potentiated the anti-inflammatory role of hypothermia at 24 h.

Macrophage Inflammatory Peptide (MIP1α) is produced by activated astrocytes in the brain (19). It is a chemo attractant and activator for polymorphonuclear cells (20, 21). Studies have shown that MIP-1α peaks 6–14 h after hypoxic ischemic injury (15, 19). MIP-1α remains elevated for 24–48 h and is undetectable at 96 h after insult (19). In our study, we observed an elevation at 24 h in female pups. However there are reports showing increase vulnerability of male neonatal rat pup brain to neuronal death following hypoxia ischemia (22–24). Some have attributed it to high exposure of testosterone (25) *in utero* in male fetus and some to difference in activation of apoptotic pathway (24, 25). We did not see a significant change in MIP 1α in male pups (at 24 h). We demonstrated an attenuation of MIP 1α at 24 h in the presence of both hypothermia and JWH 133, suggesting an anti-inflammatory effect.

Regulated on activation, normal T cell expressed and secreted (RANTES) has shown induction 24h−14 days after hypoxic ischemic injury (15, 26, 27). It acts on monocytes and T lymphocytes (26). We demonstrate upregulation of RANTES following HIE and JWH 133. In presence of hypothermia alone or both hypothermia and JWH 133, there was a significant decline in its levels at 24 h. This further supports the idea that JWH 133 is enhancing the neuroprotective effect of hypothermia.

Consistent with a previous report by Zhang et al. (28), we demonstrate an upregulation in CB2 receptors at 8 and 24 h intervals. This upregulation was significant with passage of time (i.e., from 8 to 24 h). This is important as the CB2 receptor is the site of action for JWH 133. Female pups showed a stronger response for CB2 receptor expression. Gender difference in expression of receptors has not been reported previously. CB2 receptors are reported to reside in peripheral inflammatory cells (16, 29) and resting microglial (30, 31) cells in CNS. There are reports of upregulation of receptors on macrophages and activated microglial cells in the CNS following inflammation (31). The invasion and activation of immune cells in the CNS following HIE could account for upregulation of CB2 receptors seen in our study. Hypothermia with or without JWH 133 opposes this upregulation of receptors. This suggests that hypothermia and JWH 133 are acting by different mechanisms.

In our study, we observed a significant decrease in apoptotic cells at 24 h following HIE in presence of JWH 133 alone and in conjunction with hypothermia. Cannabidiol has shown similar effects *in vitro* (13) and in a piglet model of HIE (14). Our study is the first to test JWH 133, a selective CB2 agonist, and its neuroprotective role in HIE.

Our study results were limited by the small sample size, as well as utilization of whole brain homogenateas opposed to specific regions. We did not observe any significant change in IL-6, TGF β, or IL-10 levels between the groups at 8 and 24 h. It would be worth looking at more time points, as we could have possibly missed the peak time point for these mediators. MIP 1α and RANTES are elevated much later in the inflammatory cascade so we do not see significant changes at 8 h. As this was a pilot study, whole brain homogenates were tested for immunologic and receptor changes. It is possible that there were significant alterations in some of these markers in regions of the brain more susceptible to hypoxic injury (i.e., certain layer of the cortex) which were missed when combined with less damaged regions in the whole brain sample. Further studies are warranted to examine specific regional changes as well as to establish the timeline of ischemic inflammatory damage in this model. Additionally, JWH 133 treatment resulted a significant decrease in apoptotic bodies in TUNEL experiments. It would be worth exploring the anti-apoptotic pathway as a mechanism of action of CB2 receptor agonists in future experiments.

## Data Availability Statement

The datasets for this study can be available on request by contacting the authors.

## Ethics Statement

The animal study was reviewed and approved by Institutional Animal Care and Use Committee (IACUC, MWU 2665) of Midwestern University.

## Author Contributions

BG: development of hypothesis, research and grant proposal, performed laboratory experiments, data analysis, and interpretation. PP: research mentor and advisor, guiding BG in project development. RD: research coordinator, assist with project development, and grant proposal. MH: carried out laboratory experiments, data generation, analysis, and interpretation of results. SB: oversight and data analysis and interpretation of results. AG: oversight of basic science laboratory at Midwestern.

### Conflict of Interest

The authors declare that the research was conducted in the absence of any commercial or financial relationships that could be construed as a potential conflict of interest.

## Abbreviations

HIE: Hypoxic ischemic encephalopathy
HI: Hypoxia ischemia
IL-6: Interleukin 6
TNFα: Tumor necrosis factor alpha
MIP-1a: Macrophage inflammatory protein 1 alpha
RANTES: Regulated on activation, normal T cell expressed and secreted
TGFβ: Transforming growth factor beta
TUNEL: Terminal deoxynucleotidal transferase dUTP nick end labeling
IL-10: Interleukin 10
NICHD: National Institute of Child Health and Human Development
PND: Post natal day.
